# Determinants of extended door-to-needle time in acute ischemic stroke and its influence on in-hospital mortality: results of a nationwide Dutch clinical audit

**DOI:** 10.1186/s12883-019-1512-2

**Published:** 2019-11-04

**Authors:** Laurien S. Kuhrij, Perla J. Marang-van de Mheen, Renske M. van den Berg-Vos, Frank-Erik de Leeuw, Paul J. Nederkoorn, on behalf of the Dutch Acute Stroke Audit consortium, H. F. Lingsma, G. J. de Borst, A. G. W. van Norden, M. M. Eysink Smeets, L. A. M. Aerden, C. L. Alblas, F. de Beer, H. P. Bienfait, A. E. Boon, S. Bor, A. M. H. P. Boreas, I. Bronner, R. Brouns, P. J. A. M. Brouwers, F. Brugman, M. L. Dane, P. S. S. Fransen, H. M. A. van Gemert, A. E. L. van Golde, M. T. de Graaf, L. Hani, A. M. H. G. van der Heijden, P. H. Hilkens, J. B. M. ten Holter, S. W. de Jong, L. J. Kapelle, K. Keizer, R. Keunen, R. P. Kloppenborg, A. J. M. Kok, L. Koops, N. D. Kruyt, F. E. de Leeuw, H. Lövenich, G. J. Luijckx, E. Maasland, I. Miedema, P. J. Nederkoorn, S. Persoon, E. W. Peters, T. C. van der Ree, A. D. Rozeman, R. Saxena, S. van Schaik, E. L. L. M. de Schryver, W. J. Schuiling, E. S. Schut, J. E. A. Staals, X. Stalpers, H. Tjeerdsma, J. H. van Tuijl, S. E. Vermeer, M. C. Visser, I. van den Wijngaard, M. S. G. van Zagten, S. A. Zylicz

**Affiliations:** 1 Department of Neurology, Amsterdam University Medical Center, location Academic Medical Center, Meibergdreef 9, 1105 AZ Amsterdam, the Netherlands; 2 Dutch Institute for Clinical Auditing, Rijnsburgerweg 10, 2333 AA Leiden, the Netherlands; 3 Department of Biomedical Data Sciences, Leiden University Medical Center, Albinusdreef 2, 2333 ZA Leiden, the Netherlands; 4 Department of Neurology, OLVG, Jan Tooropstraat 164, 1061 AE Amsterdam, the Netherlands; 5 Department of Neurology, Radboud University Medical Center, Houtlaan 4, Nijmegen, 6525 XZ the Netherlands; 6 Donders Center of Medical Neuroscience, Donders Institute for Brain, Cognition and Behaviour, Geert Grooteplein Zuid 10, 6526 GA Nijmegen, the Netherlands

**Keywords:** Stroke, Intravenous thrombolysis, Quality improvement

## Abstract

**Background:**

Intravenous thrombolysis (IVT) plays a prominent role in the treatment of acute ischemic stroke (AIS). The sooner IVT is administered, the higher the odds of a good outcome. Therefore, registering the in-hospital time to treatment with IVT, i.e. the door-to-needle time (DNT), is a powerful way to measure quality improvement. The aim of this study was to identify determinants that are associated with extended DNT.

**Methods:**

Patients receiving IVT in 2015 and 2016 registered in the Dutch Acute Stroke Audit were included. DNT and onset-to-door time (ODT) were dichotomized using the median (i.e. extended DNT) and the 90th percentile (i.e. severely extended DNT). Logistic regression was performed to identify determinants associated with (severely) extended DNT/ODT and its effect on in-hospital mortality. A linear model with natural spline was used to investigate the association between ODT and DNT.

**Results:**

Included were 9518 IVT treated patients from 75 hospitals. Median DNT was 26 min (IQR 20–37). Determinants associated with a higher likelihood of extended DNT were female sex (OR 1.17, 95% CI 1.05–1.31) and admission during off-hours (OR 1.12, 95% CI 1.01–1.25). Short ODT correlated with longer DNT, whereas longer ODT correlated with shorter DNT. Young age (OR 1.38, 95% CI 1.07–1.76) and admission to a comprehensive stroke center (OR 1.26, 1.10–1.45) were associated with severely extended DNT, which was associated with in-hospital mortality (OR 1.54, 95%CI 1.19–1.98).

**Conclusions:**

Even though DNT in the Netherlands is short compared to other countries, lowering the DNT may be achievable by focusing on specific subgroups.

## Background

Stroke is one of the leading causes of disability and mortality in the world [1]. Intravenous thrombolysis (IVT) with recombinant tissue plasminogen activator (rtPA) is an established intervention for patients with acute ischemic stroke (AIS). IVT increases the odds of independent functioning. With every minute the treatment is started earlier, the higher the odds of a good outcome: ‘time is brain’ [2]. When IVT is administered within 90 min after onset of symptoms, the number needed to treat (NNT) is 4.5 which is increased to a NNT of 9 between 91 and 180 min [3]. The door-to-needle time (DNT), the time from presentation of patient with symptoms at the hospital to the start of IVT, can therefore be used to evaluate the quality of the acute stroke care provided by each hospital [4]. Lowering the median DNT is an essential goal for quality improvement and is therefore used worldwide in audits for this particular purpose.

In the Netherlands, the Dutch Acute Stroke Audit (DASA) registers the median DNT as a process indicator to allow for comparison between hospitals. The median DNT is relatively short in the Netherlands compared to other countries [5]. Given that reduction in time to treatment increases the probability of a good outcome, it remains crucial to identify which patients have a longer than median DNT. The aim of this study is to identify patient- and clinical characteristics associated with (severely) extended DNT from a large nationwide registry. Secondary objectives were to identify factors associated with a delay in onset-to-door time (ODT) and to assess the impact of extended DNT on in-hospital mortality.

## Methods

### Patients and data

The DASA is a nationwide, registry-based, prospective cohort study in which data are collected of patients with AIS and hemorrhagic stroke admitted to one of the 81 hospitals participating in the registry since 2014 [5]. The DASA is a clinical audit managed by the Dutch Society of Neurology, a nationwide professional association for neurologists, and facilitated by the Dutch Institute for Clinical Auditing (DICA). It utilizes indicators to measure the quality of care and provides a national benchmark. For the present study, all consecutive patients with AIS who received IVT registered in 2015 and 2016 in the DASA were included. At this time, pre-notification by ambulance and computed tomography (CT) of the brain as primary imaging modality was considered standard of care. AIS was defined as infarction proven on imaging with a deficit exceeding 24 h. Excluded were patients with transient ischemic attack (TIA) or infarction as a complication of cerebral venous thrombosis. Under Dutch law, no informed consent or ethical approval was required for this study.

### Definitions

DNT was defined as the difference between door-time and needle-time. The definition of the door-time was the time at which the patient was presented at the emergency room with a stroke. In case the patient was already admitted to the hospital, the time of examination by the neurologist was used as the door-time. Needle-time was defined as the time when the (bolus of) rtPA was given. In 575 patients (5.4%) the DNT was unknown and therefore these were excluded from the analysis. For this study, two different cut-off points were used for analysis: ‘extended DNT’ and ‘severely extended DNT’. Extended DNT was defined as a DNT above the median, as the median DNT is used as a quality indicator of acute stroke care. Severely extended DNT was determined as a DNT above the 90th percentile. These cut-off points were chosen for two purposes: to investigate whether there is a difference in determinants associated with both cut-off points and to compare these determinants to studies in other countries where the cut-off point was around an hour corresponding with the 90th percentile of this study [6–8]. The upper limit of the DNT is set to 270 min, consistent with the timeframe of 4.5 h in which the IVT is indicated. Similarly, the upper limit of the ODT was set at 270 min.

ODT was defined as the difference between onset-time and door-time. Onset-time was defined as the time when signs and symptoms of stroke occurred, as reported by the patient or an observer. In case the patient had a stroke during sleep, or the patient could not recall the time when symptoms began, the onset time was defined as unknown. The ODT was unknown in 545 patients (5.3%). Therefore these patients were excluded from the analysis. Similar to the cut-off points for DNT, ODT cut-off points were set at median (extended ODT) and the 90th percentile (severely extended ODT). A comprehensive stroke center was specified as a stroke center in which intra-arterial thrombectomy can be performed following IVT. Primary stroke centers are those performing IVT only.

Admission during off-hours was specified as admission outside office hours from Monday until Friday, i.e. between 18:00 and 8:00 h, or on Saturday or Sunday. In-hospital mortality was defined as death during admission at the hospital.

### Statistical analysis

First, we used descriptive statistics to test for differences in patient characteristics, hospital factors as well as outcomes between 2015 and 2016, given the known decrease in DNT over time. ODT and DNT were reported as medians with interquartile ranges (IQR). Chi-square tests were used to test for differences in dichotomous variables. Mann-Whitney U test and Kruskal-Wallis tests were used to test for differences in age and ODT/ DNT respectively as these factors were not normally distributed.

Secondly, we performed logistic regression analysis to identify possible determinants for (severely) extended DNT and (severely) extended ODT. Univariate and subsequently multivariable logistic regression analysis was performed for both outcomes. The following possible determinants for extended or severely extended DNT were tested: age (continuous, in years), sex (female/male), ODT (continuous, in minutes), admission to comprehensive stroke center (yes vs no), admission during off-hours (yes vs no) and year of inclusion (2015 vs 2016). The determinants selected for identification of determinants associated with (severely) extended ODT were similar to the DNT analysis. The severity of signs and symptoms of the stroke (using a National Institute of Health Stroke Scale (NIHSS)-score) was not registered in the DASA during the study period. Continuous variables were first plotted to check for possible non-linearity. If so, points at which the relations changed were used to classify into categories. Factors with a *p*-value of 0.1 or lower in univariate analysis were included in the multivariable regression. Factors were added using forward selection and interaction between factors was investigated. The correlation between ODT and DNT was further analyzed by a generalized linear model using a natural spline. A heatmap was added to provide a graphical representation of data to visualize the density of individual DNTs for each time point. In this heatmap, the grey color indicates a low density whereas the light blue indicates higher density.

Lastly, possible determinants associated with in-hospital mortality were tested. Continuous variables (i.e. age, ODT and DNT) were first plotted to check for non-linearity and categorized by change in association. Similar logistic regression analysis was used to determine the association between DNT and in-hospital mortality, adjusted for included determinants.

R studio version 3.4.3 was used for statistical analysis.

## Results

In total, 55,860 patients with AIS from 75 Dutch hospitals were registered in the DASA in 2015 and 2016. Of these patients, 10,638 received IVT (19%). After exclusion of patients with unknown ODT (*n* = 837) and DNT (*n* = 575), 9518 could be included in the current analyses (Table 1). The mean age was 71 years and 54% were males. Median ODT was 71 min and did not significantly change over the years. Thirty-two percent of patients were admitted to a comprehensive stroke center. The percentage of patients admitted during off-hours did not change over time (*p* = 0.64). Median DNT decreased from 27 min in 2015 to 25 min in 2016 (*p* < 0.001). In-hospital mortality in this study group reduced from 6.2 to 5.6% although this was not statistically significant (*p* = 0.29).

**Table 1:** Baseline characteristics

	Total	2015	2016	*p*-value
Number of patients	9518	4814	4704	
Number of hospitals	75	75	74	
Patient characteristics				
Age in years (mean,sd)	71.0 (13.7)	70.9 (13.9)	71.0 (13.6)	0.77
Women (n, %)	4297 (46.0%)	2207 (46.4%)	2090 (45.5%)	0.39
Median onset-to-door time (IQR)	71.0 (45–119)	71.0 (45–119)	71.0 (45–119)	0.89
Hospital factors				
Admission to comprehensive stroke center	3079 (32.3%)	1619 (33.6%)	1460 (31.0%)	0.01
Admission during off-hours	4696 (49.2%)	2358 (49.0%)	2328 (49.5%)	0.64
Median door-to-needle time (IQR)	26.0 (20–37)	27.0 (20–37)	25.0 (19–35)	< 0.001
Outcome measure				
In-hospital mortality	561 (5.9%)	296 (6.2%)	265 (5.6%)	0.29

### Factors influencing the door-to-needle time

Extended DNT was thus defined as DNT above 26 min and severely extended DNT above 55 min. The impact of different determinants on (severely) extended DNT are shown in Table 2. Factors independently associated with an increased likelihood of an extended DNT were: female sex (OR 1.17, 95% CI 1.05–1.31), admission during off-hours (OR 1.12, 95% CI 1.01–1.25) and inclusion in 2015 versus 2016 (OR 1.33, 95% CI 1.14–1.35). An ODT of three hours and above had a significantly reduced likelihood of an extended DNT (OR 0.75, 95% CI 0.61–0.91).

**Table 2:** Effect of patient- and clinical determinants on door-to-needle time, expressed in odds ratio’s (with 95th confidence intervals) for different cut-off points

		Door-to-needle time			
		Univariate analysis		Multivariable analysis	
Cut-off point in minutes		Median	90th percentile	Median^a^	90th percentile^b^
		26	55	26	55
Age (years)					
	< 50	1.19 (1.01–1.40)^c^	1.42 (1.11–1.81) ^c^	1.16 (0.98–1.37)	1.38 (1.07–1.76) ^d^
	50–75	Reference			
	> 75	1.12 (0.98–1.27)	1.18 (0.95–1.45)	1.11 (0.97–1.27)	1.21 (0.97–1.49)
Female sex		1.21 (1.11–1.32) ^c^	1.08 (0.94–1.24)	1.17 (1.05–1.31)^d^	–
Onset-to-door time (minutes)					
< 40		1.10 (0.99–1.23)	1.42 (1.20–1.66) ^c^	1.15 (1.00–1.33)	1.37 (1.11–1.69) ^d^
	40–180	Reference			
	> 180	0.76 (0.65–0.88) ^c^	0.40 (0.28–0.57) ^c^	0.75 (0.61–0.91) ^d^	0.46 (0.28–0.56) ^d^
Admission to comprehensive stroke center		0.94 (0.86–1.03)	1.27 (1.10–1.46) ^c^	–	1.26 (1.04–1.50) ^d^
Admission during off-hours		1.15 (1.06–1.25) ^c^	1.08 (0.95–1.24)	1.12 (1.01–1.25) ^d^	–
Year of inclusion					
	2015	1.24 (1.14–1.34) ^c^	0.97 (0.85–1.11)	1.33 (1.14–1.35) ^d^	–
	2016	Reference			

^a^Adjusted for age, female sex, onset-to-door time, admission during off-hours and year of inclusion. ^b^Adjusted for age, onset-to-door time and admission to comprehensive stroke center. ^c^P-value below 0.1. ^d^P-value below 0.05

Factors that increased the likelihood on severely extended DNT were slightly different: age below 50 years (OR 1.38, 95% CI 1.07–1.76), admission to comprehensive stroke center (OR 1.26, 95% CI 1.04–1.50) and short ODT (OR 1.37, 95% CI 1.11–1.69). Like with an extended DNT, an ODT of three hours and above was associated with a reduced likelihood of a severely extended DNT (OR 0.46, 95% CI 0.28–0.56).

**Fig. 1:**
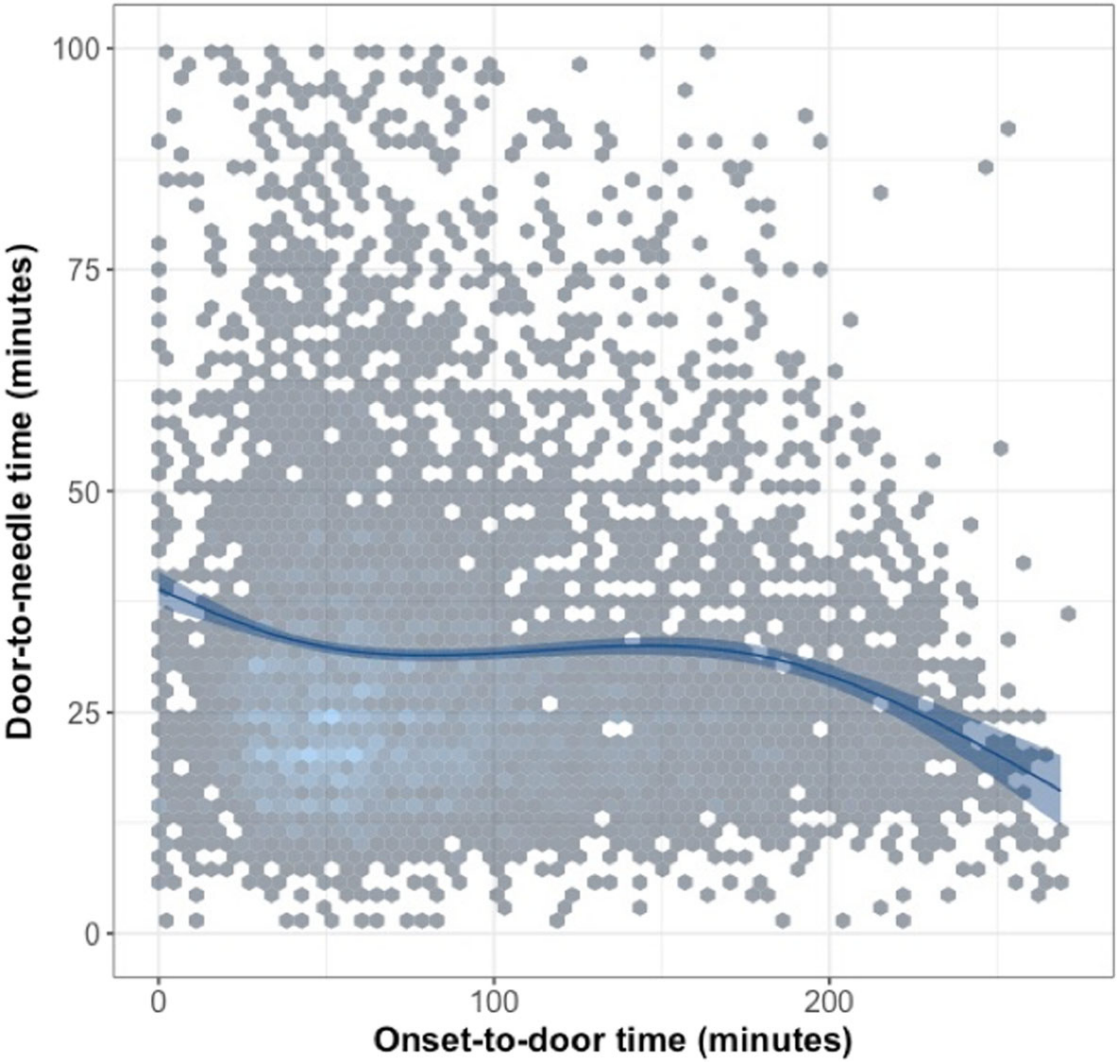
Association between onset-to-door time and door-to-needle time using a linear model with regression line and 95% CI. A heatmap was added

In Fig. 1, the correlation between ODT and DNT is visualized. It appears that patients with a short ODT have a higher DNT than patients with a long ODT. The median DNT is 27 (IQR 20–40) in patients with an ODT of 40 min or shorter. Conversely, in patients with an ODT of three hours or higher, the median DNT is 25 (IQR 18–33).

### Factors influencing the onset-to-door time

Extended ODT was defined as ODT above 71 min and severely extended ODT above 175 min. The following factors were independently associated with extended ODT: age above 75 years (OR 1.38, 95% CI 1.26–1.50), female sex (OR 1.16, 95% CI 1.06–1.26) and admission during off-hours (OR 1.12, 95% CI 1.03–1.21) (Table 3). For severely extended ODT, these determinants were similar with the addition of admission at a comprehensive stroke center (OR 1.25, 95% CI 1.08–1.44).

**Table 3:** Effect of patient- and clinical factors on onset-to-door time, expressed in odds ratio’s (with 95th confidence intervals) for different cut-off points

		Onset-to-door time			
		Univariate analysis		Multivariable analysis	
Cut-off point in minutes		Median	90th percentile	Median^a^	90th percentile^b^
		71	175	71	175
Age (years)					
	< 50	0.94 (0.80–1.10)	0.98 (0.73–1.28)	0.93 (0.80–1.10)	0.93 (0.69–1.23)
	50–75	Reference			
	> 75	1.40 (1.29–1.52)^c^	1.29 (1.12–1.49) ^c^	1.38 (1.26–1.50)^d^	1.27 (1.10–1.47) ^d^
Female sex		1.23 (1.13–1.33) ^c^	1.21 (1.05–1.39) ^c^	1.16 (1.06–1.26) ^d^	1.16 (1.01–1.33) ^d^
Admission to comprehensive stroke center		0.98 (0.90–1.06)	1.27 (1.10–1.46) ^c^	–	1.25 (1.08–1.44) ^d^
Admission during off-hours		1.11 (1.02–1.20) ^c^	1.21 (1.05–1.38) ^c^	1.12 (1.03–1.21) ^d^	1.18 (1.03–1.36) ^d^
Year of inclusion					
	2015	1.00 (0.92–1.08)	1.02 (0.89–1.17)	–	–
	2016	Reference			

^a^Adjusted for age, female sex and admission during off-hours. ^b^Adjusted for age, female sex, admission to comprehensive stroke center and admission during off-hours. ^c^P-value below 0.1. ^d^P-value below 0.05

### Effect of door-to-needle time on in-hospital mortality

Severely extended DNT (i.e. 55 min or higher) had a higher odds of in-hospital mortality in patients receiving IVT (OR 1.54, 95% CI 1.19–1.89) after adjustment for other factors. Other factors increasing the in-hospital mortality rate were age above 75 years (OR 3.45, 95% CI 2.86–4.19) and admission to comprehensive stroke center (OR 1.39, 95% CI 1.56–1.66), as shown in Table 4.

**Table 4:** Effect of patient and clinical factors on in-hospital mortality

		In-hospital mortality			
		Univariate analysis		Multivariable analysis	
		OR(95%CI)	*p*-value	OR (95% CI)	*p*-value
Age (years)					
	< 50	0.18 (0.06–0.43)	< 0.01	0.17 (0.05–0.41)	< 0.01
	50–75	Reference			
	> 75	3.53 (2.90–4.31)	< 0.01	3.45 (2.86–4.19)	< 0.01
Female sex		1.33 (1.12–1.58)	< 0.01	1.06 (0.89–1.27)	0.51
Admission to comprehensive stroke center		1.32 (1.10–1.57)	< 0.01	1.39 (1.56–1.66)	< 0.01
Admission during off-hours		1.09 (0.92–1.29)	0.34	–	–
Year of inclusion					
	2015	0.91 (0.77–1.08)	0.27	–	–
	2016	Reference			
Onset-to-door time					
	< 180	Reference			
	> = 180	0.88 (0.63–1.19)	0.42	–	–
Door-to-needle time					
	< 55	Reference			
	> = 55^a^	1.48 (1.15–1.88)	< 0.01	1.54 (1.19–1.98)	< 0.01

^a^Corresponding with the 90th percentile

## Discussion

The present study has identified factors that are associated with a higher DNT in this nationwide Dutch registry. Female sex and admission during off-hours were independently associated with a DNT above the overall median, which was 26 min. Age below 50 years, admission to a comprehensive stroke center and short ODT (ODT of 40 min or faster) were independently associated with a severely extended DNT (55 min). A severely extended DNT was associated with a higher likelihood of in-hospital mortality.

When comparing to international studies, the median door-to-needle time in the Netherlands is a lot shorter than in other countries [9–11]. In the present study, we showed that the median DNT was 27 min in 2015 which was reduced to 25 min in 2016 with a narrowing of the distribution indicating that patients are not only treated faster but also with less variability. Multiple explanations can be provided for the already short and still decreasing DNT. From 2008 the Dutch Society of Neurology, has provided a national guideline for acute stroke treatment that urged to implement a high-urgency stroke code, including pre-arrival notification by paramedics, written protocols of acute stroke care and ensuring availability of imaging modalities before the arrival of the patient [12]. At the time of the study, the primary imaging modality was computed tomography (CT). This high-urgency stroke code is known to reduce DNT [13–16], and it has been implemented throughout the Netherlands. Secondly, the median DNT for each hospital is made transparent yearly to the public, healthcare institute and health care insurers, and is used for benchmarking. This has encouraged the local optimization of care to decrease DNT [17, 18].

One of the strengths of this study is that by using such a large number of patients from a nationwide on-going registry, we are able to identify factors to allow for further improvement to establish an even shorter DNT. The factors identified in this study associated with extended DNT were female sex and admission during off- hours. Earlier studies have shown that females have a higher likelihood of extended DNT than men [19]. The current hypothesis is that women present with different neurological symptoms than men, such as pain and altered consciousness, which has a wider differential diagnosis would make diagnosis more difficult [20], similar to the sex difference in presention of acute coronary syndrome. More research has to be done to explain this sex disparity. Admission during off-hours is likely a delaying factor for IVT, implying that the acute stroke service is not as prompt during off-hours, similar to which was found in several earlier studies [8, 21]. This may be due to reduced staff or the limited availability of resources. Alternatively there may be an overrepresentation of severely affected patients during off-hours as minor stroke patients may decide to wait until the next day. In patients with severely extended DNT, age below 50 years and admission to a comprehensive stroke center were independently associated factors. In patients below 50 years of age, there could be a higher prevalence of stroke mimics making it more time consuming to make the correct diagnosis. Comprehensive stroke centers are more prone to receive the more severely affected and possibly complicated patients, which therefore could be an explanation for the increased likelihood of severely extended DNT whereas it was not associated with extended DNT. Another finding was that in patients whose ODT was longer than three hours IVT has been administered faster, which makes sense as IVT can otherwise not be given as the timewindow for treatment is up to four and a half hours. Additionally, physicians may take more time for diagnosis in patients who present very early after stroke onset. This finding is undesirable because, as mentioned earlier, the sooner the patient is treated, the higher the odds of a good outcome. Factors associated with an extended ODT were age above 75 years, female sex and admission during off-hours. We hypothesize that older patients are more often living alone, especially in case of women surviving their male partners, and that during off-hours patients are either asleep, are reluctant to seek medical help in time or experience barriers in reaching out to medical assistance. In severely extended ODT patients, admission to a comprehensive stroke center was an additional factor. We hypothesize that when a patient is almost outside the time window for treatment with IVT but could qualify for intra-arterial thrombectomy, they are presented in comprehensive stroke centers with a relatively longer ODT due to longer travel time.

In patients with AIS treated with IVT, in-hospital mortality decreased during the registry, although this was not statistically significant. Severely extended DNT showed to have a higher odds of death in these selected patients (OR 1.54), concurrent with the understanding that the earlier IVT is administered, the better the outcome. However, an alternative explanation could be that the medical status of these patients was so severe that it was contemplated whether these patients should receive IVT. ODT had no statistically significant effect on in-hospital mortality, but this could be due to the selection of patients that arrive at the hospital in time and are therefore eligible for treatment. Another factor associated with a higher in-hospital mortality rate was admission to a comprehensive stroke center. We believe that this could be explained by the fact that in comprehensive stroke centers more patients with more severe strokes will be presented which we could not adjust for sufficiently. The NIHSS score, indicating the severity of stroke, was in that period not yet registered in the DASA in that period. In more recent years, the NIHSS was added to the registry.

There are limitations to this study. Unmeasured clinical factors could explain part of the relation between the determinants and odds ratios of (severely) extended DNT. Examples of these covariates not included in this study are the NIHSS-score, other medical comorbidities (e.g. uncontrolled hypertension) and inpatients versus admitted at the emergency room could impact DNT. The NIHSS-score indicating the severity of stroke, could not be included as previously mentioned. Other research has shown that patients with a lower NIHSS score have a higher DNT [22]. Before 2017, the NIHSS score was only registered as part of trials, not on a national level and therefore was not registered in the DASA at that time. We do believe that admission at a comprehensive stroke center could be used as a proxy as patients with more severe strokes are more likely to be admitted to a comprehensive stroke center so that we will have caputered part of the effect related to the severity of stroke. However, in the absence of the NIHSS score, we could not verify this.

## Conclusions

This Dutch comprehensive stroke audit with real-world data has identified factors that are independently associated with a (severely) extended DNT. Considering that a reduction of time to treatment increases good outcome, these factors are necessary to identify. Female patients and patients admitted during off-hours have a higher odds for a DNT above the median, i.e. 26 min. Young patients and those admitted to a comprehensive stroke center are more likely to have a DNT above the 90th percentile, i.e. 55 min, and appear to have a higher in-hospital mortality rate. Having identified these factors and established its effect on in-hospital mortality, further reduction of the DNT can be established by creating awareness and focusing on these patient subgroups.

## Data Availability

The datasets analysed during the current study are not publicly available because the source of the data is a natiowide clinical audit, but are (partly) available from the Clinical Audit Board of the Dutch Acute Stroke Audit on reasonable request.

## Abbreviations

AIS: Acute ischemic stroke
CT: Computed tomography
DASA: Dutch Acute Stroke Audit
DICA: Dutch Institute for Clinical Auditing
DNT: Door-to-needle time
IVT: Intravenous thrombolysis
NIHSS: National Institute of Health Stroke Scale
NNT: Number needed to treat
ODT: Onset-to-door time
TIA: Transient ischemic attack
